# Progress in Understanding Brain Injury After Cardiopulmonary Bypass in Cardiac Surgery: A Narrative Review

**DOI:** 10.31083/RCM50277

**Published:** 2026-08-21

**Authors:** Si Zhang, Jianing Hao, Weijie Fan, Bo Liu, Wei Li, Li Wen, Dong Zhang

**Affiliations:** ^1^Department of Radiology, Xinqiao Hospital, Army Medical University, 400037 Chongqing, China; ^2^Department of Radiology, 987th Hospital of Joint Logistics Support Force of Chinese People’s Liberation Army, 721015 Baoji, Shaanxi, China

**Keywords:** cardiopulmonary bypass, cardiac surgery, brain injury, neuroprotection, precision medicine

## Abstract

The application of cardiopulmonary bypass (CPB) has significantly advanced cardiovascular surgery. However, CPB-associated brain injury remains a critical complication that affects patient prognosis and quality of life. Overt stroke occurs in approximately 1–5% of cases, while postoperative delirium affects 20–50% of patients, and perioperative neurocognitive disorders are reported in 10–40%, depending on the diagnostic criteria used. This narrative review provides a comprehensive summary of recent research progress on CPB-associated brain injury in cardiac surgery, along with a critical appraisal of the supporting evidence. While the main focus is on adult patients, various pediatric conditions are also discussed. We review the evolution of CPB technology; the classification and clinical burden of brain injury; detailed pathophysiological mechanisms, including hemodynamic injury, microembolism, systemic inflammatory response, and blood–brain barrier disruption; current cerebral protection strategies advances in personalized cerebral perfusion. Furthermore, special consideration is given to genetic susceptibility (such as apolipoprotein E (*APOE*) polymorphisms), and pediatric cardiac surgery. A systematic screening of high-quality literature from the past five years highlights the challenges that continue to impede clinical translation, despite progress in neuromonitoring and minimally invasive CPB technologies. Future research directions include the application of artificial intelligence and real-time cerebral autoregulation monitoring to support the transition from empirical medicine to precision, individualized cerebral protection.

## 1. Introduction

The aging population and rising incidence of cardiovascular disease have led to an increasing number of patients undergoing cardiac surgery under cardiopulmonary bypass (CPB). Despite continuous improvement in surgical techniques, anesthesia management, and cardiopulmonary bypass equipment, postoperative neurological complications remain a major factor affecting early mortality and long-term quality of life [1]. The clinical spectrum of brain injury is broad, ranging from catastrophic events such as overt stroke to more subtle forms of perioperative neurocognitive disorders (PND) and delirium. The latter is often clinically underappreciated, yet it significantly impacts the patients’ ability to return to society [2]. Current neuroprotective strategies are largely based on population averages and are not precisely tailored to individual pathophysiological status, leading to heterogeneity in protective efficacy.

Unlike systematic reviews that follow strict methodological protocols, this narrative review aims to provide a broad, integrative overview of CPB-associated brain injury. We highlight the key mechanisms, critically evaluate existing protection strategies, and identify promising directions for future cerebral protection strategies in clinical practice. Being a narrative review, this article does not follow PRISMA guidelines or conduct a formal risk-of-bias assessment. The literature search was performed using the PubMed, Scopus, and Web of Science databases up to March 2026, with a focus on publications from the past decade and especially the last five years. The search terms included combinations of “cardiopulmonary bypass”, “brain injury”, “neuroprotection”, “cerebral autoregulation”, “inflammation”, “microemboli”, “postoperative cognitive dysfunction”, and “perioperative neurocognitive disorders”. Reference lists from key articles were also manually reviewed. No strict inclusion/exclusion criteria or quantitative synthesis was applied, and the selection of articles was guided by the authors’ expertise and the goal of providing an integrative overview of major advances and controversies in the field. Throughout this review, we explicitly comment on the strength of the available evidence, distinguishing findings derived from large clinical trials, smaller observational studies, animal models, or preliminary reports. Where evidence is conflicting or insufficient, we highlight these gaps to guide future investigation.

## 2. The Historical Evolution of CPB and Its Role in Cerebral Protection

### 2.1 Early Exploration and the First Clinical Application

CPB technology forms the cornerstone of modern cardiovascular surgery. Looking back on its history, the early development of this technology was fraught with challenges and risks. In the mid-20th century, Gibbon and his colleagues successfully performed the first atrial septal defect repair using CPB, marking the beginning of the era of open-heart surgery. However, early CPB equipment was rudimentary, with bubble oxygenators being the mainstay. These oxygenators facilitated gas exchange by bringing blood into direct contact with oxygen. While they solved the oxygenation problem, they also generated a large number of microbubbles and caused substantial damage to blood cells, leading to a high rate of neurological complications. Due to the limited understanding of cerebral physiology at the time, cerebral protection relied mainly on the metabolic suppression afforded by deep hypothermia, with a lack of systematic strategies for microemboli filtration or inflammation control. Despite the rudimentary nature of early technology, CPB became established as an essential technique in cardiac surgery. This prompted researchers to begin investigating the impact of CPB on multiple organ systems, including the brain [3].

### 2.2 Key Technological Advances and Their Significance for Cerebral Protection

Progress in biomedical engineering has led to revolutionary advances in CPB equipment, greatly enhancing cerebral safety. The first major advance was the widespread adoption of membrane oxygenators. Compared to traditional bubble oxygenators, membrane oxygenators use a semi-permeable membrane for gas exchange, significantly reducing the blood-gas interface and thereby minimizing blood cell damage and protein denaturation caused by blood-gas contact [4]. A comparative study found the incidence of intraoperative retinal microemboli was 100% in patients using bubble oxygenators, compared with only 47% in those using membrane oxygenators. Moreover, the area of retinal non-perfusion was significantly smaller in the membrane oxygenator group (*p* < 0.01), directly demonstrating the effectiveness of membrane oxygenators in reducing microembolic load [5]. The maturation of hypothermia research represents another milestone in cerebral protection. Deep hypothermic circulatory arrest (DHCA) provides a bloodless surgical field for complex aortic procedures by dramatically reducing the cerebral metabolic rate. In pediatric cardiac surgery, deep hypothermia combined with surface cooling was once widely applied. Despite its considerable risks, this approach expanded the surgical indications for low-birth-weight infants at the time [6]. Additionally, the introduction of emboli filtration systems (particularly arterial line filters) effectively captured thrombi, fat droplets, and gaseous emboli generated during CPB, further reducing cerebral embolic events [7]. The concept of goal-directed perfusion (GDP) has emerged in recent years, marking a shift in perfusion management from an empirical approach toward physiology-guided practice. GDP emphasizes the precise adjustment of flow and pressure based on the patient’s body surface area, temperature, and metabolic demands, with the aim of avoiding both cerebral hypoperfusion and hyperperfusion injury [8].

### 2.3 Current Trends

Current CPB technology is moving toward miniaturization, improved biocompatibility, and intelligent integration. Minimally invasive extracorporeal circulation (MiECC) systems reduce tubing length and priming volume, thereby decreasing hemodilution and the surface area exposed to foreign materials, which in turn alleviates systemic inflammatory response syndrome (SIRS). Expert consensus indicates that MiECC has potential for ameliorating SIRS in adult cardiac surgery [9]. Additionally, the application of miniaturized circuits in pediatric congenital heart surgery has shown initial safety and feasibility, offering new possibilities for reducing brain injury in infants and young children [10]. Another notable trend is the integration of artificial intelligence (AI) and big data. The use of AI to analyze large volumes of intraoperative data holds promise for the real-time optimization of perfusion parameters and risk prediction, propelling CPB management into the era of precision medicine [11,12].

## 3. Classification and Clinical Burden of CPB-Associated Brain Injury

### 3.1 Classification of Brain Injury

CPB-associated brain injury is classified into two main types based on clinical presentation and imaging results: Type I injury (overt brain injury) and Type II injury (covert brain injury). Type I injury mainly includes ischemic stroke, hemorrhagic stroke, and fatal brain injury. This type typically presents with obvious neurological deficits such as hemiparesis, aphasia, or impaired consciousness, and can be clearly identified through imaging modalities such as computed tomography (CT) or magnetic resonance imaging (MRI). Type II injury is more insidious, presenting primarily as PND and delirium [13]. PND involves decline across multiple cognitive domains, including memory, attention, and executive function. It is often not detected until weeks or even months after discharge, yet it profoundly affects the patients’ ability to perform daily activities and return to work. In addition, a diffuse form of brain injury known as encephalopathy often manifests as confusion and altered mental status. This injury may be related to metabolic disturbances or cumulative microembolic burden [14].

### 3.2 Risk Factors

The development of CPB-associated brain injury results from the interplay of multiple factors. Patient-related factors include advanced age, hypertension, diabetes, prior history of stroke, carotid artery stenosis, and susceptibility genes for cerebrovascular disease such as the *APOE* ε4 allele (see Section 7.2 for details). Surgical factors involve dislodgement of plaque from the aorta during cannulation or clamping (atheroembolism), prolonged CPB duration, extended deep hypothermic circulatory arrest time, and microcirculatory insufficiency caused by non-pulsatile perfusion during surgery. Despite advances in surgical techniques, the incidence of brain injury remains high in aortic arch procedures, with embolism, inadequate cerebral blood flow, and inflammation shown to be the primary contributing factors [15]. In addition, significant intraoperative hemodynamic fluctuations—such as prolonged periods where mean arterial pressure falls below the lower limit of cerebral autoregulation—are also important independent risk factors for postoperative neurological complications [16].

### 3.3 Clinical Burden

CPB-associated brain injury imposes a substantial clinical burden. The reported incidence of overt stroke after conventional on-pump cardiac surgery ranges from 1% to 5%, with rates approaching 10% in high-risk aortic arch procedures or in patients with substantial aortic atheroma. Postoperative delirium occurs in 20–50% of patients, while PND have a highly variable reported incidence of 10–40%, depending heavily on diagnostic criteria and the timing of assessment. Overt stroke markedly increases postoperative mortality and disability rates, prolongs intensive care unit (ICU) and total hospital stay, and consumes additional healthcare resources. Even what is classified as covert brain injury has been linked to long-term cognitive decline, though the causal relationship remains debated and may reflect preexisting cerebral vulnerability [2]. Studies indicate that brain injury is closely linked to postoperative changes in mental status and may serve as a risk factor for long-term cognitive decline and dementia [17]. Epidemiological data further confirm that postoperative delirium and cognitive dysfunction remain significant concerns, with recent studies presenting refined risk stratification models [18]. In a clinical study from China of patients undergoing open-heart surgery with CPB, several patients developed psychiatric symptoms postoperatively, and the incidence of cardiac and renal dysfunction was also relatively high. These findings suggest that CPB exerts synergistic effects across multiple organ systems, and comprehensive nursing care along with early intervention is essential for improving outcomes [19]. Therefore, reducing the incidence of brain injury is not only necessary for improving patient quality of life, but also for alleviating the socioeconomic burden.

## 4. Mechanisms of CPB-Associated Brain Injury

### 4.1 Hemodynamic Injury: Hypoperfusion and Hyperperfusion

Stable cerebral blood flow is a prerequisite for maintaining neurological integrity. During CPB, factors such as non-pulsatile flow, temperature changes, and alterations in blood pressure management strategies can all lead to significant fluctuations in cerebral perfusion. Hypoperfusion injury typically occurs when the intraoperative mean arterial pressure (MAP) falls below the patient’s lower limit of cerebral autoregulation, resulting in cerebral ischemia and hypoxia. Conversely, hyperperfusion injury occurs when the MAP exceeds the upper limit of autoregulation, potentially leading to cerebral edema or hyperperfusion syndrome. Conventional blood pressure management often relies on uniform fixed thresholds (e.g., MAP 50–60 mmHg), but this approach overlooks individual variability. Studies have suggested that personalized blood pressure targets—as determined by the individual’s cerebral autoregulatory status—may better maintain the cerebral oxygen supply-demand balance and reduce postoperative complications compared with uniform targets [20]. Additionally, microcirculatory disturbances during CPB represent another important mechanism. Even when the macrovascular perfusion pressure appears adequate, reduced erythrocyte deformability and microthrombus formation can still lead to hypoxia at the tissue level [21].

### 4.2 Microembolic Events

Microembolism is one of the core mechanisms underlying CPB-associated brain injury, encompassing gaseous emboli and particulate emboli (thrombi, fat droplets, platelet aggregates). Although membrane oxygenators and arterial filters have significantly reduced the microembolic burden, microemboli remain difficult to eliminate entirely. Intraoperative surgical maneuvers—such as aortic cannulation, clamping, and anastomosis—are the primary sources of emboli formation. In patients with severe aortic atherosclerosis, in particular, dislodged plaque fragments can readily cause large-vessel embolization. At the microscopic level, tiny gaseous emboli and blood cell aggregates generated within the CPB circuit, while not causing immediate major stroke, can lead to diffuse microinfarcts and neuroinflammation, ultimately resulting in cognitive decline [22].

### 4.3 Systemic Inflammatory Response and Neuroinflammation

The systemic inflammatory response syndrome triggered by CPB plays a critical role in brain injury. Contact between blood and foreign surfaces activates the complement system (both classical and alternative pathways), generating anaphylatoxins (such as C3a and C5a) that in turn lead to neutrophil activation and aggregation [23]. These activated leukocytes release large quantities of inflammatory mediators (such as interleukin-6 (IL-6) and tumor necrosis factor-alpha (TNF-α)) that not only damage vascular endothelium, but can also cross the compromised blood-brain barrier (BBB) to enter the central nervous system. Within the central nervous system, microglia—the resident immune cells—are activated by peripheral inflammatory signals and transform into the M1 phenotype, releasing reactive oxygen species (ROS) and pro-inflammatory cytokines that cause synaptic damage and neuronal apoptosis [24]. Microglial activation has been shown to play an indispensable role in neuroinflammation associated with CPB and deep hypothermic circulatory arrest, and interventions that target this pathway could therefore represent a novel approach for cerebral protection [25]. Moreover, inflammation exacerbates coagulation disturbances, creating a vicious “inflammation-coagulation” cycle that further aggravates brain injury [26].

### 4.4 Disruption of the Blood-Brain Barrier

The integrity of the BBB is essential for maintaining central nervous system homeostasis. CPB-related inflammatory storms, oxidative stress, and hemodynamic fluctuations can all disrupt the BBB. Disruption of endothelial tight junctions, pericyte dysfunction, and degradation of the basement membrane are the main pathological manifestations of BBB breakdown. Once the permeability of the BBB increases, toxic substances, autoantibodies, and inflammatory cells from the peripheral circulation can more readily infiltrate the brain parenchyma, triggering direct neurotoxicity and secondary edema [27]. Research indicates that BBB dysfunction is a key pathological mechanism in many central nervous system disorders, setting off a cascade of events such as neuroinflammation and oxidative stress that ultimately exacerbate brain injury [28]. In models of systemic inflammation, as the severity and duration of inflammation increase, solute permeability across the BBB rises and lymphocyte infiltration intensifies, eventually leading to encephalopathy-like clinical presentations [29]. Therefore, preservation of BBB integrity is a crucial component of cerebral protection strategies.

### 4.5 Impaired Cerebral Autoregulation

Cerebral autoregulation refers to the ability of cerebral blood flow to remain relatively constant despite fluctuations in perfusion pressure within a certain range. CPB, hypothermia, anesthetic agents, and preexisting cerebrovascular disease can all impair autoregulatory function. When autoregulation is compromised, cerebral blood flow becomes pressure-passive. This means that even small fluctuations in blood pressure can lead to dramatic changes in cerebral blood flow, greatly increasing the risk of cerebral ischemia or hemorrhage [30]. Real-time monitoring of autoregulatory status and adjusting the perfusion pressure accordingly represents a key step toward individualized cerebral protection. Current monitoring techniques rely predominantly on transcranial Doppler or near-infrared spectroscopy to calculate cerebral oximetry indices or pressure reactivity indices, thereby identifying the optimal perfusion pressure range for each patient [31].

### 4.6 Temperature Management and the Metabolism-Oxidative Stress Axis

Hypothermia reduces cerebral oxygen consumption by lowering the cerebral metabolic rate, making it a classic cerebral protective strategy. However, temperature management is a double-edged sword. Excessively low temperatures can increase blood viscosity, impair microcirculatory perfusion, and contribute to reperfusion injury during rewarming. Metabolic demand rises abruptly during rewarming, and if oxygen delivery fails to keep pace, substantial amounts of ROS are generated. This triggers oxidative stress, which can attack lipids, proteins, and DNA, leading to neuronal necrosis or apoptosis [32]. Different temperature levels have varying effects on cerebral autoregulation, and moderate hypothermia may preserve cerebral autoregulatory function better than deep hypothermia. Whether remote ischemic preconditioning combined with moderate hypothermia offers superior cerebral protection compared with deep hypothermia alone is currently being investigated [33].

### 4.7 Summary of Evidence Hierarchy

Evidence regarding the mechanisms of CPB-associated brain injury spans multiple levels, from basic animal models to clinical observational studies, but the strength of support varies considerably across mechanisms [34]. Strong clinical evidence supports the role of macroembolism, particularly atheroembolism resulting from aortic manipulation, and global cerebral hypoperfusion that occurs when mean arterial pressure falls below the lower limit of cerebral autoregulation [35]. Moderate clinical evidence—derived largely from observational studies employing transcranial Doppler and neuroimaging—implicates cumulative gaseous and particulate microemboli in postoperative cognitive decline; however, a definitive dose–response relationship has yet to be established in large‑scale randomized trials. The involvement of systemic inflammation and blood‑brain barrier disruption is well documented in animal models and is supported by human biomarker studies (e.g., elevated IL‑6 and S100B), but direct evidence that anti‑inflammatory interventions improve neurological outcomes in unselected cardiac surgery populations remains limited and inconsistent [36]. Emerging or speculative mechanisms include specific microglial activation pathways (primarily characterized in rodent CPB models), the comparative utility of different cerebral autoregulation monitoring indices (such as the pressure reactivity index vs. the cerebral oximetry index), and the clinical relevance of genetic modifiers such as *APOE* polymorphisms. Although the latter have been replicated across multiple cohorts, their effect size is modest and their utility for perioperative risk stratification remains unproven at present [37]. Explicit recognition of this evidence hierarchy is necessary to avoid overstating preliminary findings and to prioritize translational research efforts. Heterogeneity across studies persists, and certain relationships—for example, the precise quantitative link between microembolic load and domain‑specific cognitive decline—still require validation through high‑quality, prospective clinical trials.

## 5. Cerebral Protection Strategies

### 5.1 Preoperative Risk Stratification

The identification of high-risk patients before surgery is the first step in implementing cerebral protection. This includes a thorough assessment of the patient’s cerebrovascular status (such as carotid ultrasound, computed tomography angiography), baseline cognitive function, and genetic background. For patients with severe aortic atherosclerosis, preoperative aortic imaging to evaluate plaque burden can guide the choice of cannulation site (such as switching to axillary artery cannulation), thereby reducing the risk of stroke [38]. Additionally, the use of scoring systems to predict the risk of postoperative cognitive decline can help formulate individualized perioperative management plans. Although genetic screening is not yet routine practice, studies indicate that *APOE* ε4 carriers have a higher susceptibility to brain injury [39], suggesting that it may become part of future risk stratification measures.

### 5.2 Intraoperative Multimodal Monitoring and Perfusion Management

Single monitoring modalities are unable to meet the demands of precise cerebral protection, opening the way for multimodal monitoring. Near-infrared spectroscopy (NIRS) monitors cerebral oxygen saturation and allows early detection of cerebral desaturation events, guiding perfusionists to adjust flow or blood pressure. NIRS was reported to have significant value in preventing brain injury in children with congenital heart disease [40]. Its parameters correlate with laboratory markers of brain injury, thus helping to establish effective cerebral protection strategies. Electroencephalography (EEG) is used to monitor electrical brain activity and promptly detect epileptiform discharges or ischemic suppression. In particular, EEG is a sensitive indicator for assessing electrical silence and brain functional status during deep hypothermic circulatory arrest [41]. Transcranial Doppler (TCD) enables real-time monitoring of microembolic signals, helping surgeons adjust their maneuvers to reduce embolus dislodgment [42]. Based on feedback from these monitoring data, the implementation of goal-directed perfusion management (e.g., maintaining a relative decline in cerebral oxygen saturation of <20% from baseline, or an absolute value no lower than 50–60%) is recommended by several guidelines [43]. Recent reviews have similarly emphasized the importance of integrating cerebral autoregulation monitoring, perfusion pressure targets, and hypothermic management in cardiac and aortic surgeries [44].

### 5.3 Pharmacological Neuroprotection

Although numerous drugs targeting the mechanisms of brain injury have been investigated, clinical translation has thus far yielded only limited results. Aside from anesthetic agents such as isoflurane and propofol that may have preconditioning or postconditioning effects, there is currently a lack of specific neuroprotective drugs [45]. Anti-inflammatory drugs, antioxidants (e.g., N-acetylcysteine), and antiepileptic drugs have all been studied to varying degrees, but the results have been inconsistent. For example, while high-dose corticosteroids may reduce inflammation, they also increase the risk of infection and hyperglycemia. Some experimental drugs have shown promise in reducing hippocampal injury in animal models of deep hypothermic circulatory arrest (e.g., the cannabinoid receptor agonist WIN55,212-2), but clinical evidence is still lacking [46]. Chinese expert consensus has mentioned the application of integrated traditional Chinese and Western medicine in cerebral protection, but it was also noted that pharmacological treatment was currently limited to an adjunctive role only [47]. Overall, the optimization of physiological parameters (blood pressure, temperature, and glucose) is supported by stronger clinical evidence than single-drug interventions.

### 5.4 Postoperative Neurorehabilitation and Biomarker Monitoring

Early neurological assessment in the postoperative period is critical. The use of biomarkers such as S100B, neuron-specific enolase (NSE), or glial fibrillary acidic protein (GFAP) for early diagnosis can help to initiate interventions in a timely manner [48]. Nevertheless, it is important to acknowledge the limitations of currently used biomarkers. The specificity of S100B is compromised by its expression in extracranial tissues such as adipose tissue, chondrocytes, and bone, and its levels can be elevated by hemolysis or fat embolism during CPB. The expression of ubiquitin carboxy‑terminal hydrolase L1 (UCH‑L1) is more neuron‑specific than S100B. However, UCH‑L1 remains an investigational marker in adult cardiac surgery, and its extracranial sources and the influence of renal function remain incompletely characterized. Consequently, neither S100B nor UCH‑L1 has been validated for routine clinical decision‑making in CPB management, and the interpretation of these biomarkers should always be combined with clinical assessment and imaging findings. For patients who develop cognitive impairment or delirium, a multidisciplinary rehabilitation plan should be implemented, including cognitive training, psychological support, and physical therapy. Furthermore, postoperative monitoring and the control of risk factors for brain injury—such as atrial fibrillation, low cardiac output, and infection—should be continued in order to prevent secondary brain injury.

### 5.5 A Pragmatic Clinical Framework for Cerebral Protection

Based on the evidence reviewed, we propose a three‑phase framework to guide perioperative decision‑making.

(1) Preoperative phase: Systematic risk stratification should include assessment of aortic plaque burden (by epiaortic or computed tomography angiography) in patients aged >65 years or with known vascular disease, baseline cognitive screening (e.g., Montreal Cognitive Assessment), and, in selected cases, discussion of *APOE *genotyping as a risk modifier—though not yet for routine clinical use.

(2) Intraoperative phase: Multimodal monitoring incorporating near‑infrared spectroscopy (NIRS) and, when available, transcranial Doppler or processed electroencephalography, combined with individualization of mean arterial pressure targets based on cerebral autoregulation monitoring (rather than fixed thresholds). Any NIRS desaturation should trigger a structured checklist (blood pressure, pump flow, arterial oxygen content, carbon dioxide tension, and inspection for malposition of cannulas).

(3) Postoperative phase: Routine delirium screening (e.g., Confusion Assessment Method for the ICU) at least twice daily; avoidance of hypotension (mean arterial pressure <65 mmHg in high‑risk patients); cautious use of sedatives with long half‑lives; and early mobilization. Biomarkers such as S100B or neuron‑specific enolase remain adjunctive research tools at present, lacking the specificity and prospective validation required for routine clinical guidance [36].

## 6. Individualized Cerebral Perfusion: Toward Precision Management

### 6.1 Real-Time Assessment of Cerebral Autoregulation

Although traditional perfusion management often relies on fixed blood pressure thresholds, this approach overlooks individual differences in cerebral autoregulatory function. The basis of individualized cerebral perfusion lies in using real-time monitoring techniques to assess each patient’s autoregulatory status. By analyzing the correlation between arterial blood pressure and different physiological signals, various autoregulation indices can be calculated. The pressure reactivity index (PRx) is derived from the moving correlation coefficient between mean arterial pressure (MAP) and intracranial pressure (ICP). The cerebral oximetry index (COx), based on near-infrared spectroscopy (NIRS), is calculated as the moving correlation between regional cerebral oxygen saturation (rSO_2_) and MAP. Transcranial Doppler (TCD)-derived indices, such as Mx (correlation between mean flow velocity and cerebral perfusion pressure) and Mxa (correlation between mean flow velocity and MAP), reflect autoregulation through changes in cerebral blood flow velocity. Contemporary reviews have summarized the clinical methods for monitoring autoregulation, including NIRS-based indices, TCD-derived metrics, and ICP-based pressure reactivity assessment, reinforcing the validity of this approach [49]. These indices, despite all being used to assess cerebral autoregulation, are derived from different physiological signals and should not be used interchangeably or assumed to share the same interpretation or thresholds. When these index values are low, this indicates that cerebral blood flow lies within the range of intact autoregulation. When the index values rise, it suggests that cerebral blood flow has become pressure-passive, and the corresponding blood pressure value represents the optimal perfusion pressure or the lower limit of autoregulation. Based on this principle, clinicians can maintain the patient’s real-time blood pressure within an individualized optimal range, thereby maximizing the safety of cerebral perfusion [50]. However, PRx-guided management has limitations that prevent its routine clinical use, including the need for invasive ICP monitoring, specialized software, and high-quality signal processing that are yet to be standardized in CPB practice. Moreover, evidence from prospective studies demonstrating improved outcomes with PRx-guided management remains limited.

### 6.2 Individualized Perfusion Parameters

Beyond blood pressure, the CPB flow rate, temperature management, blood gas strategies, and hematocrit all require individualized settings. For example, in elderly patients or those with cerebrovascular disease, maintaining a higher perfusion pressure and flow rate may be more beneficial for preventing cerebral hypoperfusion. Conversely, for patients who are at risk of bleeding, appropriately lowering the flow rate and blood pressure may reduce this risk. Key aspects of individualized management with regard to temperature are the selection of an appropriate depth of hypothermia based on surgical complexity and duration, along with strict control of the rewarming rate to avoid a sharp increase in cerebral metabolic demand that outpaces oxygen delivery. Studies have shown that moderate hypothermia combined with remote ischemic preconditioning may, in certain circumstances, provide superior cerebral protection compared to deep hypothermia [33]. This finding suggests that temperature strategies should be tailored to the individual.

### 6.3 Blood Gas Management Strategies

During CPB, blood gas management primarily follows two strategies: alpha-stat and pH-stat. The alpha-stat strategy maintains pH at approximately 7.40 based on temperature-uncorrected blood gas values (measured at 37 °C), which helps to preserve cerebral homeostasis and enzyme activity. The pH-stat strategy, in contrast, uses temperature-corrected values to maintain pH at approximately 7.40 at the patient's actual body temperature, thereby allowing a degree of hypercapnia. This increases cerebral blood flow and may facilitate more uniform brain cooling during deep hypothermia or help flush out microemboli. The choice of strategy should be guided by patient age, surgical type, and monitoring data. For instance, during deep hypothermic circulatory arrest, pH-stat is often used prior to rewarming to increase cerebral blood flow and oxygen delivery. In routine normothermic CPB, alpha-stat may be more suitable for preserving cerebral autoregulatory function [51].

### 6.4 Artificial Intelligence and Multimodal Data Integration

The large volume of intraoperative monitoring data (blood pressure, flow rate, NIRS, EEG, temperature, blood gases, etc.) exceeds the capacity for real‑time human synthesis, making AI an attractive concept. Early proof‑of‑concept studies have used machine learning, including convolutional neural networks to predict cerebral desaturation events several minutes in advance, and recurrent neural networks for postoperative delirium risk stratification using physiological time series. Computational fluid dynamics modeling based on preoperative aortic anatomy has also been used to simulate emboli trajectories, suggesting potential for surgical planning (e.g., cannula positioning) [52]. However, it is critical to emphasize that these applications remain highly preliminary. Robust clinical evidence for improved outcomes with AI‑driven perfusion management is lacking. Most reports derive from retrospective analyses or small, single‑center pilot studies without external validation. There are no standardized algorithms, data preprocessing pipelines, or intervention thresholds. Regulatory approval for closed‑loop AI systems in the operating room has not been obtained, and the “black‑box” nature of deep learning models raises concerns about interpretability and trust. Therefore, at present, AI must be viewed as an aspirational research direction rather than a clinically implementable strategy for cerebral protection [53].

## 7. Special Considerations

### 7.1 Neurological Risks in Pediatric Cardiac Surgery

The brains of pediatric patients, particularly neonates and infants, are in a rapid phase of development, making them more vulnerable to ischemia and hypoxia. Deep hypothermic circulatory arrest is widely used in complex congenital heart surgery in children, yet its long-term impact on neurodevelopment remains the subject of ongoing debate. Despite advances in surgical techniques, neonatal aortic arch surgery is still associated with varying degrees of neurodevelopmental morbidity, and the duration of DHCA correlates with neurodevelopmental outcomes [54]. However, there is also evidence suggesting that neurodevelopmental delay in children with congenital heart disease stems primarily from prenatal injury rather than being solely attributable to surgery, with functional magnetic resonance imaging (fMRI) studies indicating that minor brain injuries sustained during surgery may recover within a short period [54]. Nevertheless, intraoperative neuroprotection continues to be strongly emphasized, including the use of selective antegrade cerebral perfusion, optimization of pH-stat strategies, and close monitoring of NIRS and EEG. A survey conducted in the United Kingdom and Ireland revealed that while centers reached consensus on cooling targets (such as a nasopharyngeal temperature of 18 °C) and the use of selective antegrade cerebral perfusion, considerable variation remained in areas such as medication management, acid-base balance, and NIRS intervention thresholds. Moreover, most centers expressed a willingness to participate in clinical trials of neuroprotective strategies, reflecting the ongoing uncertainty in this field [55].

### 7.2 Genetic Susceptibility

Genetic background determines an individual’s susceptibility to CPB-associated brain injury as well as their capacity for neural repair. Among the most extensively studied genetic factors is the apolipoprotein E gene polymorphism. The *APOE* ε4 allele is a major genetic risk factor for late-onset Alzheimer’s disease and has also been linked to an increased risk of cardiovascular disease. Recent research has revealed a significant interaction between the *APOE* ε4 genotype and cardiovascular risk factors in microstructural alterations of the basal forebrain nucleus basalis of Meynert (NBM). Among carriers of *APOE* ε3/ε3 and ε3/ε4 alleles, higher cardiovascular risk is associated with increased free water signal in the NBM, suggestive of microstructural injury. In contrast, free water values remain elevated in *APOE* ε4/ε4 homozygotes, regardless of the cardiovascular risk level, and the impact of cardiovascular risk on fractional anisotropy is more pronounced than on free water [56]. These findings suggest the *APOE* ε4/ε4 genotype accelerates early microstructural changes through alterations in lipid homeostasis, disruption of neurovascular integrity, and sustained neuroinflammation, thereby increasing the risk of postoperative cognitive impairment. Additionally, the *APOE* ε2 allele has been independently associated with adverse early neurodevelopmental outcomes following infant cardiac surgery, indicating that genetic variants linked to reduced capacity for neural repair represent important risk factors [57]. In summary, there is significant potential for the incorporation of genetic background into individualized risk assessment.

## 8. Future Perspectives

Future research on CPB-associated cerebral protection will increasingly focus on precision and prevention. First, for AI and machine learning to become clinically useful, prospective multicenter trials with predefined performance metrics, standardized data collection, and transparent algorithmic architectures will be required. The current proof‑of‑concept studies must be followed by external validation, regulatory clearance, and demonstration of improved clinical outcomes (e.g., reduced delirium or stroke) rather than merely predictive accuracy. Second, risk stratification based on genetics and biomarkers may become more refined, allowing preoperative identification of high-risk populations (e.g., *APOE* ε4 carriers) and the development of targeted protective strategies, though cost‑effectiveness and ethical considerations will need careful examination. Third, the application of novel biomaterials could lead to circuit tubing and oxygenators with improved biocompatibility, but the magnitude of incremental benefit over modern membrane oxygenators and minimized circuits remains to be quantified. Fourth, for the prevention and control of microemboli, advances in real-time transesophageal echocardiography and acoustic detection technologies may improve the ability to identify and clear emboli, but these approaches remain investigational and lack standardized protocols. Finally, specific drugs that target neuroinflammation and repair of the BBB are expected to be explored further [58]. Despite promising results in animal models (e.g., inhibitors of microglial signaling pathways), translation to human perioperative neuroprotection has not yet been achieved, and rigorous dose-finding and safety studies are needed. Large-scale, multicenter randomized controlled trials remain the gold standard for validating the effectiveness of these emerging strategies. Future study designs should also place greater emphasis on long-term neurological follow-up, in particular the assessment of cognitive function and quality of life, rather than relying solely on short-term surrogate endpoints.

## 9. Discussion

This review describes the research progress to date on CPB-associated brain injury, revealing both the inherent complexity and challenges in this field. Technological improvements from bubble oxygenators to membrane oxygenators, and from deep hypothermia to minimally invasive extracorporeal circulation, have significantly reduced certain risks of brain injury, but have not entirely eliminated neurological complications. This suggests that relying solely on technical advances in equipment has limitations, and that it is essential to gain a deeper understanding of the pathophysiological mechanisms and to implement more refined management strategies.

With regard to mechanisms, we have emphasized the interplay among microemboli, systemic inflammatory response, hemodynamic fluctuations, and disruption of the BBB. Importantly, these mechanisms do not operate in isolation, but rather act as cause and effect in a mutually reinforcing cycle. For instance, microemboli can trigger local inflammatory responses, and inflammatory mediators can in turn disrupt the BBB, exacerbating cerebral edema. The additive effect of multiple mechanisms often means that single interventions are inadequate. Current evidence suggests that individualized perfusion management guided by the monitoring of cerebral autoregulation represents one of the most promising directions in recent years. This approach challenges the conventional reliance on fixed blood pressure thresholds and embodies a physiology-driven paradigm of precision medicine. However, consensus has yet to be reached within the academic community regarding how best to implement individualized management, including the selection of monitoring indicators and the definition of intervention thresholds. Further high-quality research in this field is needed.

The discussion of special populations reveals several important insights. Studies in pediatric patients suggest that our perspective should extend beyond intraoperative protection to encompass prenatal development and long-term postoperative recovery, in recognition of the impact of congenital heart disease on brain development. Genetic research, particularly findings related to *APOE* polymorphisms, provides a biological basis for understanding variability in patient outcomes. Although future neuroprotective strategies are likely to become increasingly tailored to the individual, genetic screening remains in its early stages of clinical application, and issues such as cost-effectiveness and ethical considerations require careful consideration.

This review also identified several recurrent limitations in existing research. Many clinical studies on cerebral protection strategies have relatively small sample sizes and focus primarily on short-term outcomes, such as in-hospital stroke rates or surrogate biomarkers, while follow-up data on long-term cognitive function and quality of life remain sparse. Definitions of key outcomes—particularly perioperative neurocognitive disorders and delirium—vary considerably across studies, limiting cross-trial comparability and meta-analytic efforts. Publication bias favoring positive findings may also contribute to an overly optimistic view of certain interventions. Some pharmacological agents that showed promising results in animal models failed to translate successfully in clinical trials, possibly due to species differences, timing of administration, and dosing selection. Although artificial intelligence holds theoretical promise, the “black box” nature of its algorithms, lack of external validation, and the need for regulatory approval represent hurdles that must be addressed before clinical implementation. Explicit acknowledgment of these methodological limitations is essential for interpreting the current evidence and for designing more rigorous future studies.

In summary, the prevention and management of CPB-associated brain injury constitute a systems-level endeavor that requires close collaboration among surgeons, anesthesiologists, perfusionists, and neurologists. Future efforts should shift from reactive management toward proactive prevention, and from a one-size-fits-all approach toward individualized precision management. Multimodal monitoring and AI technologies should be leveraged to construct an optimal cerebral protection framework for each patient.

## 10. Conclusion

CPB-associated brain injury represents a formidable clinical challenge in adult cardiac surgery, encompassing a broad spectrum ranging from overt stroke to subtle cognitive dysfunction. This review has detailed the multiple pathophysiological mechanisms underlying brain injury, including hemodynamic instability, microemboli, systemic inflammatory response, BBB disruption, and genetic susceptibility. Current cerebral protection strategies have achieved some progress in terms of hardware improvements (e.g., membrane oxygenators, minimally invasive circuits) and intraoperative monitoring (e.g., NIRS, EEG). However, the traditional population-based management model that relies on fixed parameters is no longer adequate to meet the demands of individualized precision medicine.

Future developments are reasonably anticipated to move toward greater precision and intelligent assistance, but this transition will require substantially more clinical evidence than is currently available. Real-time assessment of cerebral autoregulation to establish individualized perfusion parameters holds promise, supported by physiological reasoning and observational data, yet awaits confirmation in prospective randomized trials. The deep integration of multimodal data through artificial intelligence remains an aspirational goal, given the current lack of standardized, externally validated systems and regulatory approval. Risk stratification based on genetic background, together with the exploration of novel drugs targeting neuroinflammation and blood-brain barrier repair, represents an avenue for further investigation, but none of these approaches are yet ready for routine clinical adoption. Continued integration of basic mechanistic research with rigorous clinical translation will be essential to reduce the incidence of CPB-associated brain injury and to improve long-term quality of life for patients undergoing cardiovascular surgery, thereby advancing from experience-based toward evidence-based precision medicine.
